# High expression of syndecan-4 is related to clinicopathological features and poor prognosis of pancreatic adenocarcinoma

**DOI:** 10.1186/s12885-022-10128-y

**Published:** 2022-10-05

**Authors:** Yufei Zhu, Dijie Zheng, Linhan Lei, Kun Cai, Huahua Xie, Jian Zheng, Chao Yu

**Affiliations:** 1 Department of Hepatobiliary Surgery, The Affiliated Hospital of Guizhou Medical University, Guiyang, 550004 China; 2 Guizhou Medical University, Guiyang, 550004 China; 3 School of Clinical Medicine, Guizhou Medical University, Guiyang, China; 4 Guizhou Provincial Institute of Hepatobiliary, Pancreatic and Splenic Diseases, Guiyang, China; 5 Key Laboratory of Liver, Pancreas and Spleen of Guizhou Medical University, GallbladderGuiyang, China; 6 School of Basic Medical Sciences, Guizhou Medical University, Guiyang, China

**Keywords:** Pancreatic adenocarcinoma, SDC4, TCGA, GTEx, Clinicopathological feature, Survival curve, Poor prognosis, Independent risk factor

## Abstract

**Objective:**

Pancreatic adenocarcinoma (PAAD) is a leading cause of cancer-related mortality in adults. Syndecan-4 (SDC4) is involved in cancer pathogenesis. Therefore, this study aimed to explore the expression and clinical significance of SDC4 in PAAD.

**Methods:**

Differentially expressed genes (DEGs) between PAAD and normal pancreas were screened from the GTEx and TCGA databases, and the correlationship between the DEGs and prognosis were analyzed. The prognostic value of the screened SDC4, SERPINE1, and SLC2A1 was evaluated using the Kaplan–Meier curve and SDC4 was subsequently selected as the better candidate. Also, SDC4 expression was analyzed in PAAD tissues, the other risk factors affecting postoperative survival were analyzed using Cox regression analysis, and SDC4-mediated pathways enrichment was identified by GSVA and GSEA. SDC4 expression in PAAD tissues and adjacent normal tissues of selected PAAD patients was detected by RT-qPCR and immunohistochemistry. The correlation between SDC4 and clinical features was evaluated by the χ^2^ test.

**Results:**

SDC4 was highly expressed in PAAD tissues. Elevated SDC4 was correlated with reduced overall survival. SDC4 enrichment pathways included spliceosome function, proteasome activity, pentose phosphate pathway, base excision repair, mismatch repair, DNA replication, oxidative phosphorylation, mitotic spindle formation, epithelial-mesenchymal transition, and G2M checkpoints. SDC4 was elevated in PAAD tissues of PAAD patients compared with adjacent normal tissues. High SDC4 expression was related to metastatic differentiation, TNM stage, lymphatic metastasis, and lower 3-year survival rate. SDC4 was an independent risk factor affecting postoperative survival.

**Conclusion:**

SDC4 was highly expressed in PAAD and was related to clinicopathological features and poor prognosis, which might be an important index for PAAD early diagnosis and prognosis.

## Introduction

Pancreatic adenocarcinoma (PAAD) is one of the most lethal tumors with an increasing incidence, which is characterized by the presence of an abundance number of cancer-associated fibroblasts and fibrosis [1]. It was predicted as the 2^nd^ major cause of cancer deaths in some regions [2]. The risk factors for PAAD include non-modifiable factors such as age, sex, ethnicity, blood group, gut microbiota, family history, genetic susceptibility, and diabetes, and modifiable factors such as smoking, alcohol consumption, chronic pancreatitis, obesity, dietary factors, and infection [2]. At present, surgical resection is still the only theoretical treatment for PAAD [3]. However, less than 20% of PAAD patients are suitable for surgery, because most PAAD patients are diagnosed at an advanced stage and lose the opportunity of surgical treatment [4]. Despite the emergence of various chemotherapy regimens and the advances in surgical approaches, the poor prognosis of PAAD has not been improved in the last several decades, with most PAAD patients experiencing metastasis and recurrence, even after the curative resection [5]. Therefore, exploring promising biomarkers for the early diagnosis of PAAD and new therapeutic methods is needed urgently.

Syndecan-4 (SDC4) is a ubiquitously expressed, transmembrane proteoglycan bearing heparan sulfate chains, which plays roles in numerous outside-in and inside-out signaling processes, including extracellular matrix component and growth factor binding and sequestration, small GTPase Rac1 activity, protein kinase C-alpha activation, intracellular calcium level regulation, and focal adhesion kinase phosphorylation regulation [6]. SDC4 acts as a main endogenous membrane-associated receptor to regulate cell migration, cell adhesion, and cytoskeleton in tumorigenesis and progression, which can be an ideal anti-cancer therapeutic target [7]. SDC4 is closely associated with the occurrence and development of osteosarcoma, breast cancer, prostate cancer, colorectal cancer, and many other cancers [8–10]. Moreover, SDC4 is involved in pancreatic tissue repair [11]. N-syndecan expression is closely related to poor prognosis and metastasis in PAAD [12]. However, whether SDC4 can be used as a diagnostic marker of PAAD is largely unknown.

As the importance of precision medicine has been emphasized recently, genomic research is expanding from the bench discovery to the bedside diagnosis and treatment [13, 14]. Through these efforts, public databases including Genotype-Tissue Expression (GTEx), the Cancer Genome Atlas (TCGA), and other databases related to various cancer types and their genomes have been established, and these data have been actively used for research. Therefore, this study aimed to search the TCGA and GTEX databases to explore the expression and clinical significance of SDC4 in PAAD.

## Materials and methods

### Data sources: online databases

The genes related to PAAD were predicted from the public databases. Because there were few paracancerous normal samples (*N* = 4) in the TCGA PAAD database, the normal pancreatic sample data (*N* = 167) from the GTEx database were downloaded. The two groups of data were combined after removing the difference between batches to acquire 167 normal samples and 178 tumor samples.

Hypoxia-related genes were selected using these databases. The h.all.v7.1.symbols.gmt was downloaded from the GSEA official website and 200 HALLMARK_HYPOXIA-related genes were obtained.

### Screening and correlation analysis of SDC4-related proteins

STRING (Search Tool for the Retrieval of Interacting genes/proteins) database (https://string-db.org/cgi/input.pl) is an online searching biological database for exploring the interaction between proteins, which can be employed to find multiple proteins closely related to SDC4 protein and construct a protein–protein interaction (PPI) network. The PPI of hypoxia-related proteins was downloaded from the STRING website, the degree, and connectivity of the related proteins were calculated, and the hub genes were screened.

The expression matrix of differentially expressed genes was extracted and combined with clinical data. The prognosis-related genes were obtained through the Kaplan–Meier survival analysis, the target genes (SDC4, SERPINE1, and SLC2A1) that were both differentially expressed genes and prognosis-related genes were further screened, and the prognostic values of target genes on the overall survival of PAAD patients were evaluated. The expression of SDC4 in PAAD tissues was analyzed based on the GTEx and TCGA databases. The factors affecting postoperative survival were analyzed using the COX regression method. In addition, the enrichment of SDC4-mediated pathways was analyzed from GSVA and GSEA.

### Study subjects

The specimens and clinical data were collected from 72 patients with PAAD who underwent PAAD resection in Affiliated Hospital of Guizhou Medical University from January 2015 to October 2018. Clinical specimens included paraffin-embedded tumor tissues and the corresponding paracancerous tissues (more than 3 cm away from the cancer tissues). Clinical data included patient gender, age, tumor location, tumor size, tumor node metastasis (TNM) stage, histological grade, and lymph node metastasis. Inclusion criteria were as follows: patients who were pathologically diagnosed with PAAD and underwent radical surgery, without radiotherapy and chemotherapy before the operation and with complete clinical and follow-up data. Exclusion criteria were as follows: patients with incomplete clinical and follow-up data, a history of previous malignant tumors, and other complicated malignant tumors.

The enrolled patients were followed up via telephone or outpatient service to determine their tumor recurrence and survival time. The death of the patient was taken as the endpoint of follow-up observation and defined as the occurrence of the outcome event.

### Reverse transcription-quantitative polymerase chain reaction (RT-qPCR)

Total RNA was extracted from tissues using TRI reagent (Sigma-Aldrich, St Louis, MO, USA) according to the manufacturer's instructions and then reverse transcribed into cDNA using PrimeScript RT kits (Takara Bio Inc., Kusatsu, Shiga, Japan). QPCR was performed using SYBR Premix Ex Taq mix (Takara Bio Inc.) in the ViiA™ 7 Real-Time PCR System (Applied Biosystems, Foster City, CA, USA). The relative level of SDC4 normalized by the endogenous control GAPDH was calculated by the 2^−ΔΔCt^ method. The primer sequences are shown in Table 1.

**Table 1:** RT-qPCR primer sequence

Gene	Forward 5’-3’	Reverse 5’-3’
SDC4	TTGTCAATGGCAGTCTCAGC	CCTCCCGTTTCACACAACTT
GAPDH	GAAGGTGAAGGTCGGAGTC	GAAGATGGTGATGGGATTTC

### Immunohistochemistry

PAAD tissues and paracancerous tissues were fixed in 10% neutral formalin and embedded in paraffin. After sectioning, dewaxing, and rehydration, 4 μm sections were subjected to antigen retrieval. Subsequently, tissue sections were incubated with primary antibody of SDC4 (1:100, Thermo Fisher Scientific Inc., Eugene, OR, USA) at 4 °C overnight. The expression of SDC4 was evaluated from staining intensity and the percentage of positively stained cells was determined using the Image-Pro Plus (IPP) v.6.0 software. The staining intensity was divided into four grades based on the IPP score: 0 (no staining), 1 (weak staining), 2 (moderate staining), and 3 (strong staining). The percentage of staining positive cells were as follows: 0 (0), 1 (1%-25%), 2 (26%-50%), and 3 (> 50%). Total staining score = the percentage of staining positive cells × intensity score. The total score of 0–2 was defined as the low expression and the total score of 3–9 was defined as high expression.

### Statistical analysis

SPSS v.21.0 statistical software (IBM Corp., Armonk, NY, USA) and GraphPad Prism v.8.01 software (GraphPad Software Inc., San Diego, CA, USA) were employed for statistical analyses and mapping. Measurement data were expressed as mean ± standard deviation. An independent *t-*test was applied for comparisons among groups. Count data were expressed as the number of cases/percentages and tested by X^2^ test. The survival rate was calculated using the Kaplan–Meier method and the survival curve was drawn. COX regression model was adopted to analyze the factors affecting postoperative survival. *P-value of* < 0.05 was indicative of statistical significance.

## Results

### Identification of differentially expressed genes (DEGs) in PAAD and normal pancreatic tissues

**Fig. 1:**
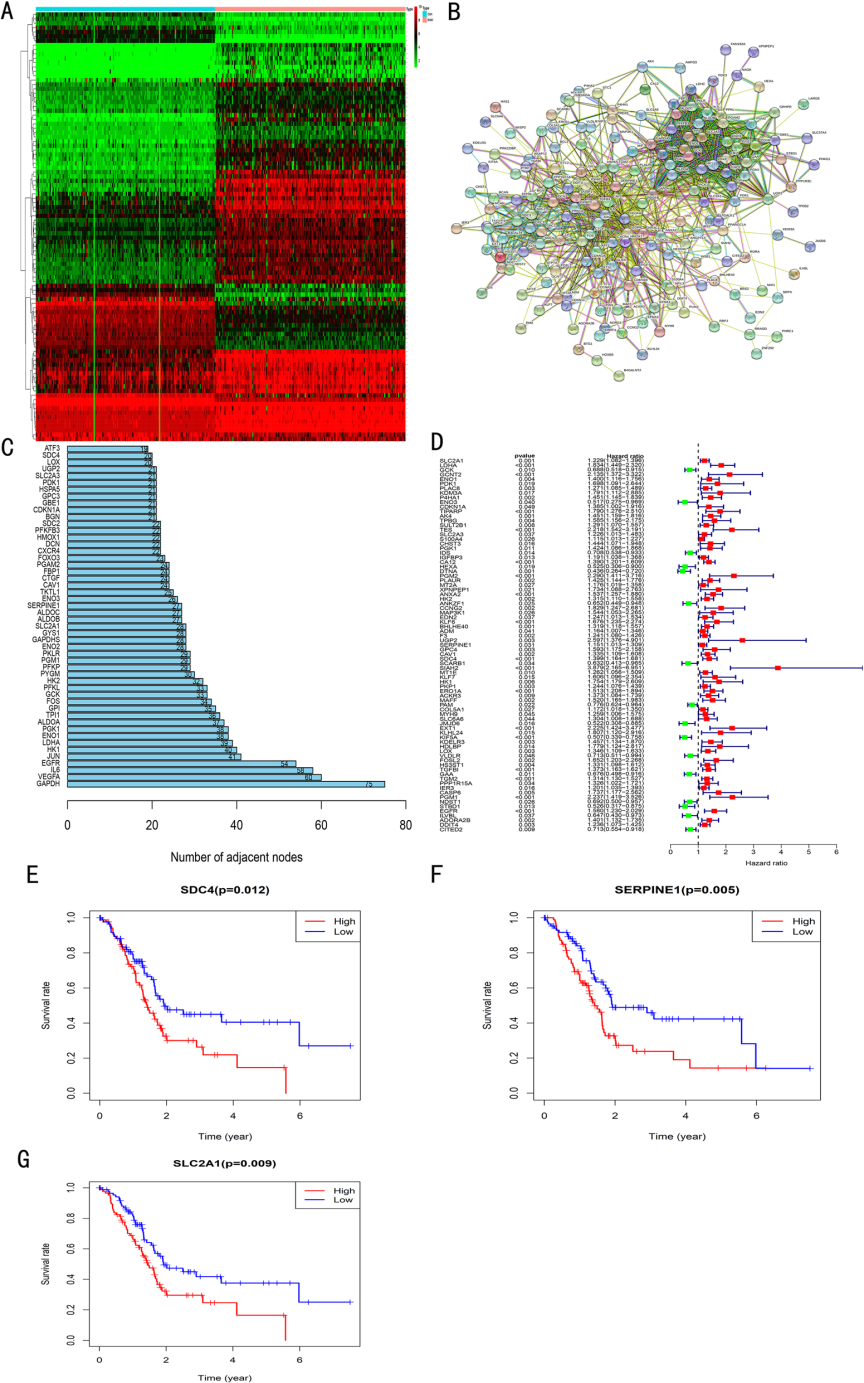
Identification of DEGs in 178 PAAD patients and 167 normal pancreatic tissues. **A** Heat map of hypoxia-related DEGs in normal pancreatic tissue and PAAD; **B** PPI network of hypoxia-related genes; **C** Calculation of Degree and connectivity in the network and screening of hub genes; **D** Expression of DEGs using limma package of R software; **E–G** Survival curve of high and low expression groups of SERPINE1, SLC2A1, and SDC4 genes in PAAD using Kaplan–Meier survival analysis

The h.all.v7.1.symbols.gmt was downloaded from the GSEA website and 200 HALLMARK_HYPOXIA-related genes were extracted. After background correction, normalization, and batch effect adjustment with fdrFilter of 0.05 and logFCFilter of 0.5 as the thresholds, 98 DEGs including 74 upregulated genes and 24 downregulated genes were screened (Fig. 1A). Then, the PPI network of hypoxia-related genes was downloaded from the STRING website (Fig. 1B). The degree and connectivity of the network were calculated and the hub genes were screened (Fig. 1C). The expression matrix of DEGs was extracted and combined with clinical data. Univariate Cox regression analysis and the Kaplan–Meier survival curve were performed to screen prognosis-related genes (Fig. 1D). The genes that were DEGs, prognosis-related genes, and the core genes from the PPI network (Degree of > 20) were screened, and 6 genes, namely SERPINE1, SLC2A1, SDC4, LOX, HK2, and CAV1 were finally identified. Subsequently, the 6 genes and PAAD were searched on PubMed using them as keywords. The search results consisted of 3 articles with SERPINE1, 3 articles with SLC2A1, 0 articles with SDC4, 66 articles with LOX, 28 articles with HK2, and 25 articles with CAV1. SDC4, SERPINE1, and SLC2A1were selected as the focus of this study since there were fewer research reports.

The 178 PAAD specimens from the TCGA database were downloaded. Because there were few normal pancreatic data in the TCGA PAAD database (*N* = 4), 167 normal pancreatic data from the GTEX database were downloaded. Regarding survival analysis, the specimens were divided into two groups, the high expression group (higher than the average gene expression) and the low expression group (lower than the average gene expression). After survival analysis of the three candidate genes, there were significant differences in the survival time of the three genes (SDC4, SERPINE1, and SLC2A1) between the two groups (*P* < 0.05) (Fig. 1E-G). However, SERPINE1 and SLC2A1 linked to PAAD were reported, while SDC4 in PAAD were not reported. Therefore, we finally selected SDC4 as the gene for the following analyses.

### Prognostic potential of SDC4 in PAAD patients

**Fig. 2:**
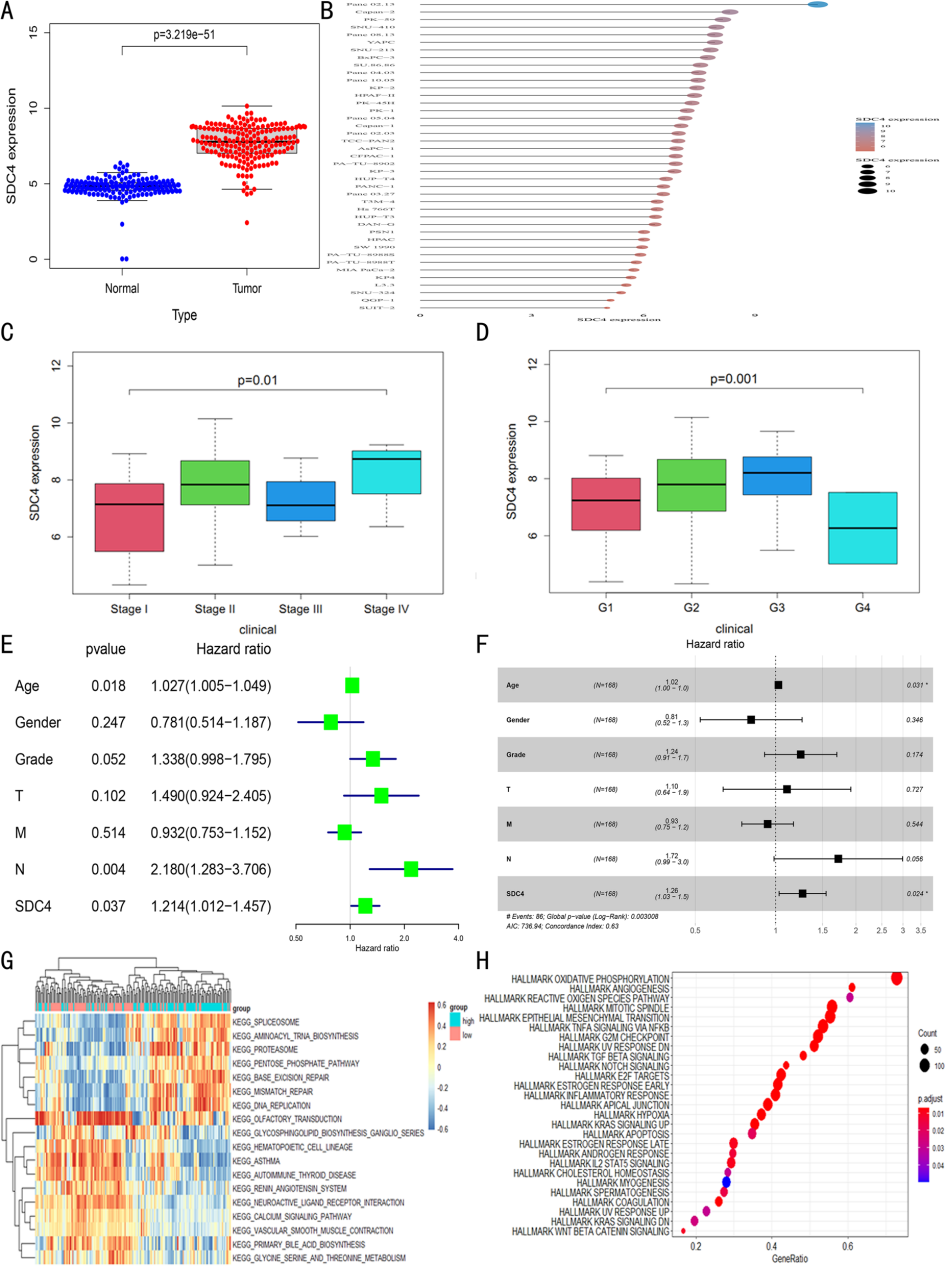
Prognostic potential of SDC4 in PAAD patients by the “survival” package in R Software. **A** The expression of SDC4 in normal and tumor tissues using Wilcox test (*P* < 0.05); **B** The expression of SDC4 in different pancreatic cancer cells; **C** The expression of SDC4 in different TNM stages using Kruskal–Wallis tests; **D** The expression of SDC4 in different Grades; **E** and **F** Univariate and multivariate COX regression analyses of the factors affecting the prognosis and survival of PAAD; **G** Heat map of the main enrichment pathways of SDC4 from GSVA method; **H** The main enrichment pathways of SDC4 were analyzed by GSEA method

The differential analysis of TCGA-PAAD cohort showed that SDC4 expression in tumor patients was increased compared with the control group (*P* < 0.01) (Fig. 2A). SDC4 was also higher in Panc 02.13 PAAD cells of a cell line (Fig. 2B). Regarding TNM staging, the SDC4 level in the stage IV group was higher than that in the stage I group (*P* = 0.01) (Fig. 2C). Regarding grade stage, SDC4 expression in the G4 grade was lower than that in the G1 grade (*P* = 0.01) (Fig. 2D). Univariate and multivariate analyses were performed for variables including age, gender, grade, TNM stage, and SDC4 expression parameters. The results revealed that age and SDC4 were independent risk factors for the overall survival of PAAD patients (*P* < 0.05) (Fig. 2E, F). GSVA analysis revealed that the enriched pathways of SDC4 mainly included spliceosome function, aminoacyl tRNA biosynthesis, proteasome activity, pentose phosphate pathway, base excision repair, mismatch repair, and DNA replication (Fig. 2G). GSEA analysis demonstrated that the enriched pathways of SDC4 mainly included oxidative phosphorylation, mitotic spindle formation, epithelial-mesenchymal transition, TNFα signaling via NFKB, and G2M checkpoint (Fig. 2H).

### SDC4 is highly expressed in PAAD tissues

**Fig. 3:**
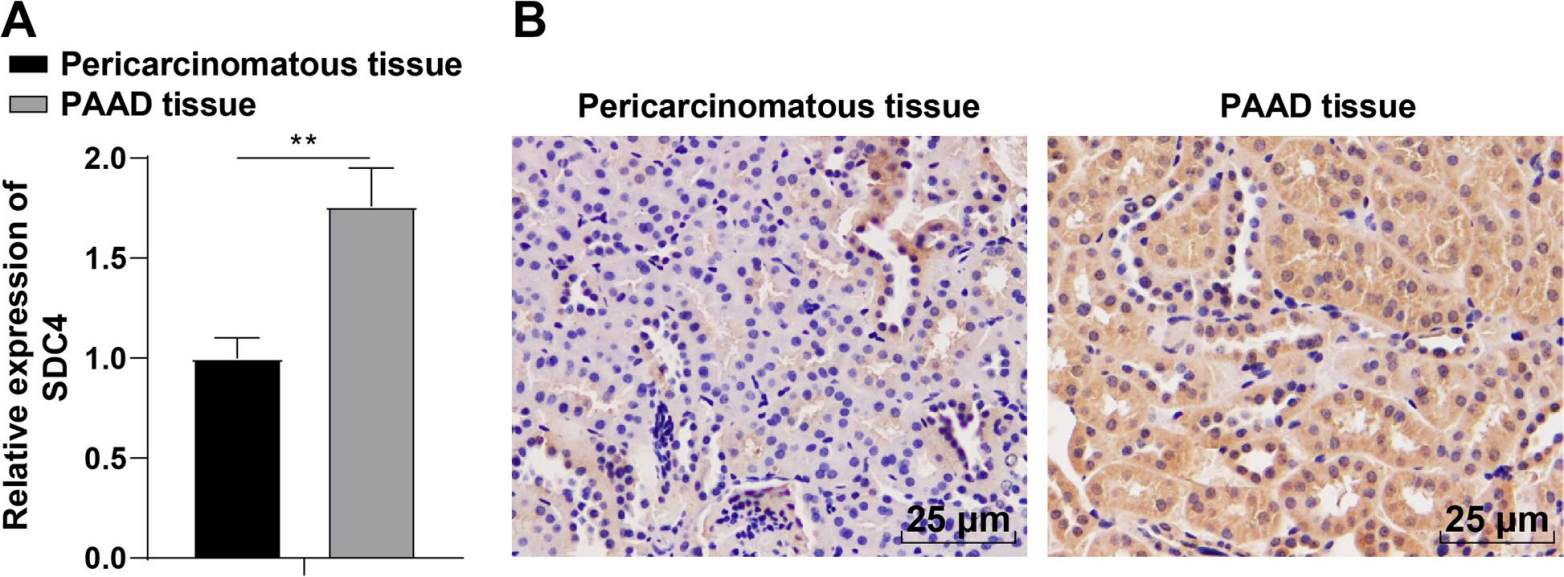
SDC4 expression in PAAD and corresponding paracancerous tissues. **A** The expression of SDC4 in PAAD and paracancerous tissues was detected by RT-qPCR; **B** The expression of SDC4 in PAAD and paracancerous tissues was detected by immunohistochemistry (mainly expressed in the cytoplasm, brown yellow particles). Independent sample *t*-test was applied for data analysis. ** *P* < 0.01

To further study the expression and clinical significance of SDC4 in PAAD, 72 patients with PAAD who underwent PAAD resection in Affiliated Hospital of Guizhou Medical University from January 2015 to October 2018 were selected. The expression of SDC4 in PAAD tissues and paracancerous normal tissues was detected by RT-qPCR, which revealed higher SDC4 expression in PAAD tissues than that in corresponding paracancerous tissues (*P* < 0.01) (Fig. 3A). The SDC4 expression in PAAD tissues and paracancerous tissues was detected by the immunohistochemical method, which showed more brownish-yellow pigments in cancer tissues than that in paracancerous tissues (Fig. 3B), indicating higher SDC4 expression in cancer tissues than that in paracancerous tissues. Among the selected patients, 42 cases showed high SDC4 expression in PAAD tissues, and the high expression rate was 58%; 7 cases showed high SDC4 expression in paracancerous tissues, and the high expression rate was 10%. Further statistical analysis by Chi-square test showed that there were significant differences in the expression of SDC4 between PAAD and corresponding paracancerous tissues (χ^2^ = 37.89, *P* < 0.001) (Table 2).

**Table 2:** Expression of SDC4 in PAAD and corresponding paracancerous tissues

Tissues	High expression cases	High expression rate (%)	**χ**^**2**^ value	*P* value
PAAD tissues	42	58	37.89	< 0.001
Paracancerous tissues	7	10		

### Correlation between SDC4 expression and clinicopathological features of PAAD patients

To study the correlation between SDC4 and clinical indexes, PAAD patients were assigned into low expression (*n* = 30) and high expression groups (*n* = 42) based on the immunohistochemical results. It was found that SDC4 expression had no significant correlation with gender, age, tumor size, and tumor location of PAAD patients (*P* > 0.05). However, the SDC4 expression had significant correlations with tumor differentiation, TNM stage, and lymph node metastasis (*P* < 0.05) (Table 3).

**Table 3:** Correlation between the expression of SDC4 and clinicopathological features of PAAD patients

		SDC4			
Clinical parameters	Case	Low expression (30 cases)	High expression (42 cases)	**χ**^**2**^ value	*P* value
Gender				0.529	0.467
Male	42	16	26		
Female	30	14	16		
Age (year)				0.196	0.658
≤ 60	31	12	19		
> 60	41	18	23		
Tumor size (cm)				0.65	0.42
≤ 3	10	3	7		
> 3	62	27	35		
Tumor location				1.754	0.185
Pancreatic head	49	23	26		
Pancreatic tail	23	7	16		
Differentiation degree				6.334	0.012
Low + middle	58	20	38		
High	14	10	4		
Tumor TNM stage				6.612	0.01
I + II	46	14	32		
III + IV	26	16	10		
Lymph node metastasis				8.316	0.004
No	29	18	11		
Yes	43	12	31		

### SDC4 is an independent risk factor for the survival of PAAD

**Fig. 4:**
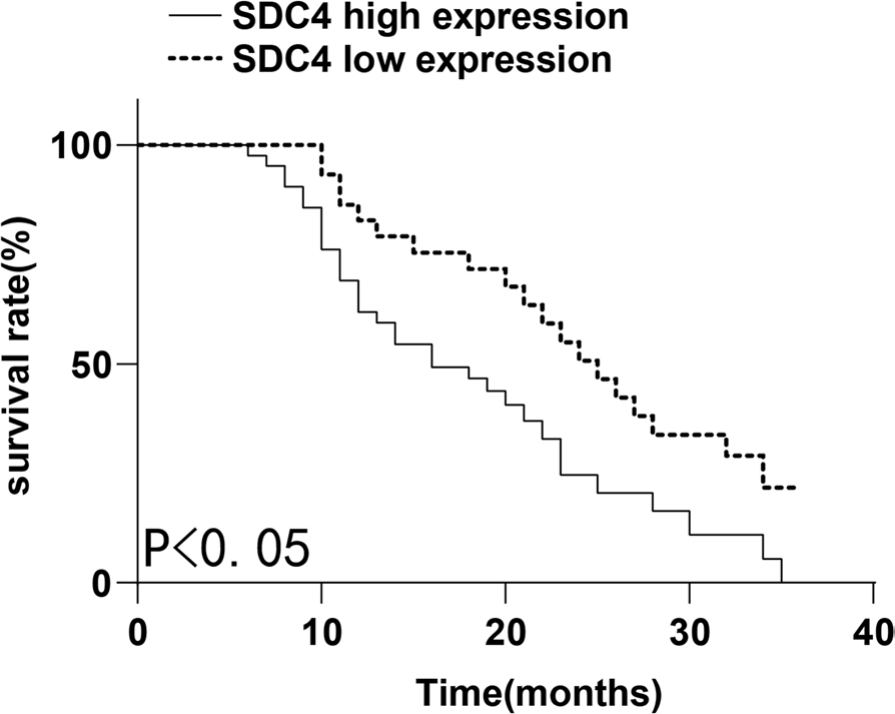
Kaplan–Meier curve of SDC4 expression and survival time

The postoperative follow-up data of 72 PAAD patients were collected and analyzed by the Kaplan–Meier survival method. The 3-year survival rate of patients with high SDC4 expression was 21.4% and the median survival time was 13 months. The 3-year survival rate of patients with low SDC4 expression was 36.6% and the median survival time was 21 months. The median survival time of patients with high SDC4 expression was 8 months shorter than that of patients with low SDC4 expression. There were significant differences in the overall survival rate between the two groups(χ^2^ = 6.789, *P* = 0.009) (Fig. 4). Moreover, COX regression analysis showed that lymph node metastasis and SDC4 expression were independent risk factors affecting the prognosis of PAAD patients (Table 4).

**Table 4:** Analysis of risk factors affecting the prognosis of PAAD patients

Independent variable	B value	SE value	Wals value	*P* value	OR value	95% CI
Differentiation degree	0.745	0.427	3.053	0.081	2.107	0.913–4.861
Tumor TNM stage	-0.524	0.372	1.983	0.159	0.592	0.286–1.228
Lymph node metastasis	0.736	0.346	4.517	0.034	2.087	1.059–4.115
SDC4	-1.064	0.342	9.693	0.002	0.345	0.177–0.674

## Discussion

PAAD is lethal cancer with a rising incidence and usually, the patients are presented at an advanced stage, which causes the 5-year survival rate of 2%-9%. Therefore, it ranks the worst among all cancers in terms of the prognostic outcomes in patients [2]. Evidence showed that SDC4 regulates cell migration, cell adhesion, and cytoskeleton development in tumorigenesis and progression, so it is regarded as a probable anti-cancer therapeutic target [7]. This study concentrated on the expression and clinical significance of SDC4 in PAAD patients and illustrated that SDC4 was highly expressed in PAAD and was related to clinicopathological features and poor prognosis, which might be an important index for PAAD early diagnosis and prognosis.

We downloaded h.all.v7.1.symbols.gmt data file from GSEA, extracted HALLMARK_HYPOXIA-related genes, and screened DEGs. After screening DEGs, prognosis-related genes, and hub genes through the PPI network, we selected SERPINE1, SLC2A1, SDC4, LOX, HK2, and CAV1for further focus. After excluding the genes that were already reported in PAAD, we finally chose SDC4 to comprehensively elucidate. SDC4 is closely associated with cancer occurrence and development including osteosarcoma, breast cancer, prostate cancer, and colorectal cancer [8–10]. Our results indicated that SDC4 expression was elevated in PAAD patients and Panc 02.13 PAAD cells. SDC4 expression in PAAD tissues was higher than that in corresponding paracancerous tissues, consistent with the high expression of SDC4 in various cancers. In brief, SDC4 was upregulated in PAAD. Elevated N-syndecan level was closely associated with the poor prognosis and metastasis in PAAD [12]. Our results revealed that SDC4 expression was upregulated in stage IV in comparison with stage I; SDC4 expression was lowered in G4 compared with G1. Importantly, univariate and multivariate analyses revealed that the age, N classification, and SDC4 expression were independent risk factors for the overall survival of PAAD patients. Existing studies highlighted the significant role of SDC4 as a potential marker for myocardial infarction [15], nonalcoholic fatty liver disease [16], and endothelial dysfunction in patients with resistant hypertension [17]. Moreover, we used GSEA and GSVA to screen the pathways associated with SDC4 enrichment, which mainly included spliceosome function, aminoacyl tRNA biosynthesis, proteasome activity, pentose phosphate pathway, base excision repair, mismatch repair, DNA replication, oxidative phosphorylation, mitotic spindle formation, epithelial-mesenchymal transition, TNFα signaling via NFKB, and G2M checkpoint. The pentose phosphate pathway, mismatch repair pathway, and oxidative phosphorylation pathway are reported to play a role in PAAD [18–20]. This study initially discovered upregulated SDC4 expression in PAAD and identified it as a risk factor for the overall survival of PAAD patients. However, there are a few studies on the expression pattern of SDC4 in PAAD. The expression of SDC4 in PAAD and its clinical significance need to be further explored.

We included 72 patients with PAAD who underwent PAAD resection in Affiliated Hospital of Guizhou Medical University from January 2015 to October 2018. This study initially discovered upregulated SDC4 expression in PAAD samples. Subsequently, we categorized the PAAD patients into the high SDC4 expression and low SDC4 expression groups and found that SDC4 expression had correlations with tumor differentiation, tumor TNM stage, and lymph node metastasis. SDC4 upregulation increased the risk of renal cell carcinoma metastasis [21]. Moreover, the 3-year survival rate of high SDC4 expression patients was 21.4%, with the median survival time of 13 months, and that of low SDC4 expression patients was 36.6%, with the median survival time of 21 months. Lymph node metastasis and SDC4 were independent risk factors affecting PAAD patient prognosis. Consistently, SDC4 overexpression in the invading tumor cells is clearly related to the progression of pathogenesis and is inversely related to the overall survival of patients with colorectal cancer [22]. Breast cancer patients with high SDC4 expression had worse overall survival [23]. However, there are a few studies on the relationship between SDC4 and PAAD clinicopathological features. This study discovered that SDC4 expression was significantly correlated with clinicopathological features of PAAD, and was an independent risk factor for the survival of PAAD patients.

In summary, this study firstly detected the DEGs between PAAD and normal pancreatic tissues and found the relationship between these DEGs and PAAD prognosis based on GTEX and TCGA databases. And the study included 72 patients with PAAD to comprehensively study the expression and clinical significance of SDC4 in PAAD. This study supported that SDC4 was highly expressed in PAAD and was related to clinicopathological features and poor prognoses, which might provide an important value to determine the prognosis of PAAD. However, the number of cases and events included in the analysis of this study is small, so it is necessary to further expand the sample size, incorporating multi-center research to further clarify the value of SDC4 in the diagnosis and prognosis of PAAD. Moreover, this study simply confirmed the expression of SDC4 in PAAD and its relationship with prognosis. Further research is required on the specific mechanism of SDC4 in PAAD.

## Data Availability

All data generated or analyzed during this study are included in this published article.
